# Reduced evoked activity and cortical oscillations are correlated with anisometric amblyopia and impairment of visual acuity

**DOI:** 10.1038/s41598-021-87545-9

**Published:** 2021-04-15

**Authors:** Hanna Julku, Santeri Rouhinen, Henri J. Huttunen, Laura Lindberg, Johanna Liinamaa, Ville Saarela, Elina Karvonen, Sigrid Booms, Jyrki P. Mäkelä, Hannu Uusitalo, Eero Castrén, J. Matias Palva, Satu Palva

**Affiliations:** 1 Neuroscience Center, Helsinki Institute of Life Science, University of Helsinki, P.O. Box 21, 00014 Helsinki, Finland; 2 BioMag Laboratory, HUS Medical Imaging Center, Helsinki, Finland; 3 Herantis Pharma Plc, Bertel Jungin aukio 1, 02600 Espoo, Finland; 4 Department of Ophthalmology, Helsinki University Hospital, Helsinki, Finland; 5 Department of Ophthalmology, Oulu University Hospital, Medical Research Center (MCR), University of Oulu, Oulu, Finland; 6 Department of Ophthalmology, Tampere University, Faculty of Medicine and Health Technology, Tampere, Finland; 7 Tays Eye Center, Tampere University Hospital, Tampere, Finland; 8 Centre for Cognitive Neuroimaging, Institute of Neuroscience and Psychology, University of Glasgow, Glasgow, UK; 9 Department of Neuroscience and Biomedical Engineering, Aalto University, Espoo, Finland; 10 Present Address: Department of Ophthalmology, Helsinki University Hospital, Helsinki, Finland

**Keywords:** Neuroscience, Diseases, Signs and symptoms

## Abstract

Amblyopia is a developmental disorder associated with abnormal visual experience during early childhood commonly arising from strabismus and/or anisometropia and leading to dysfunctions in visual cortex and to various visual deficits. The different forms of neuronal activity that are attenuated in amblyopia have been only partially characterized. In electrophysiological recordings of healthy human brain, the presentation of visual stimuli is associated with event-related activity and oscillatory responses. It has remained poorly understood whether these forms of activity are reduced in amblyopia and whether possible dysfunctions would arise from lower- or higher-order visual areas. We recorded neuronal activity with magnetoencephalography (MEG) from anisometropic amblyopic patients and control participants during two visual tasks presented separately for each eye and estimated neuronal activity from source-reconstructed MEG data. We investigated whether event-related and oscillatory responses would be reduced for amblyopia and localized their cortical sources. Oscillation amplitudes and evoked responses were reduced for stimuli presented to the amblyopic eye in higher-order visual areas and in parietal and prefrontal cortices. Importantly, the reduction of oscillation amplitudes but not that of evoked responses was correlated with decreased visual acuity in amblyopia. These results show that attenuated oscillatory responses are correlated with visual deficits in anisometric amblyopia.

## Introduction

Amblyopia is a developmental condition where the optically best-corrected visual acuity is impaired in one eye (or rarely in both eyes) even though no ocular abnormalities usually are present. It commonly develops when one of the eyes have abnormal visual input, either physical or physiological, during the sensitive period in childhood from birth to the age of 6 years^1,2^. The leading causes associated with amblyopia are misaligned eyes (strabismic amblyopia) and/or unequal refractive error (anisometropic amblyopia)^3–7^. This kind of a functional imbalance between the two eyes results in an abnormal visual experience during the critical period for visual development and induces an ocular-dominance shift of visual cortical neurons in favour of the leading eye and a loss of visual acuity in the deprived eye (amblyopia)^8^. Dysfunctions of visual perception for stimuli presented to the amblyopic eye are correlated with alterations in the underlying synaptic, neuronal, and microcircuit connectivity properties in primary and secondary visual cortices^9,10^. However, neural basis of anisometropic amblyopia especially at the systems level have still remained incompletely understood, and also morphological alterations in the visual cortex could lead to anisometropic amblyopia^10,11^.

Amblyopia is characterized by abnormal visual-performance-related functions localized to primary visual cortex, i.e., to the visual acuity measures and measures related to contrast sensitivity, which are pronounced for stimuli with high spatial frequencies^3–7^. These are differentially implicated by etiology, as anisometric amblyopia is more characterized by decreased visual acuity while decreased contrast sensitivity and stereopsis is more typical to strabismic amblyopia. Some studies furthermore show that amblyopia impacts vision not only in the amblyopic eye (AE) but also in the fellow eye (FE)^12^. In line, perceptual deficits in amblyopia can also arise from abnormal neuronal suppression between eyes^13^ and by inadequate inhibition in visual cortex between FE and AE^14^. Importantly, amblyopic patients suffer also from deficits in higher-order processing functions such as motion detection, temporal integration, peripheral vision, and capability to perceive multiple items, which cannot simply arise from deficits exclusively localized to early visual regions^5,15^. Amblyopia is hence a complex disorder and the mechanisms, the underlying neural structure and activity, behind the variety of visual deficits remain unclear.

The prevalence of amblyopia in the general population varies from 1.3 to 3.6%, and it is one of the most common causes of monocular visual impairment in adults^16,17^. Hence, understanding the neuronal basis of amblyopia in humans is important and serves the development of novel therapeutic approaches^18,19^. Many prior studies have investigated brain function alterations in amblyopia using functional magnetic resonance imaging (fMRI). These studies have shown that stimuli presented to the AE induce less activity than stimuli presented to the FE. Importantly, this apparent neuronal processing deficit is observed not only in early visual regions^13,20^ and lateral occipital cortex (LOC)^21^, but also in fronto-parietal attention networks^22^. However, fMRI has poor temporal resolution and does not reveal the spatio-temporal dynamics of neuronal activity at the sub-second time scales of perceptual processes. Electrophysiological methods, such as magneto- and electroencephalography (MEG/EEG), are useful methods for resolving the impact of brain diseases on brain activity with millisecond-resolution temporal accuracy and also good spatial accuracy when combined with source modelling^23–25^. Event-related responses (ERs) to visual stimuli have been found to be suppressed in both EEG scalp potentials^26,27^ and MEG sensor fields^28,29^ for simple visual stimuli such as gratings. Also, global motion^30^ and faces^31^ elicit reduced ERs in scalp EEG. However, because no studies have neither identified the cortical sources underlying these reduced responses nor addressed whether they are correlated with the visual acuity deficit and severity of amblyopia, the functional significance of this suppression has remained unresolved.

In healthy brains, one characteristic feature in electrophysiological activity are neuronal oscillations that reflect rhythmic modulations of neuronal excitability. Oscillations and their synchronization constitute a fundamental mechanism for establishing precise temporal relationships between neuronal responses and for coordinating neuronal processing^32,33^. Synchronized, temporally correlated, oscillations may regulate neuronal communication both because synchronization of neuronal spiking increases its post-synaptic impact^32^ and because temporally aligned excitability windows facilitate communication between neuronal assemblies^33^. In humans, local cortical oscillations are key constituents of visual processing related to feature specificity of visual information^34–40^. In contrast, abnormal oscillations and inaccuracies in temporal relationships contribute to symptoms and emergence of neurodevelopmental^23,41,42^ and neurodegenerative^24,43^ brain diseases. The significance of neuronal oscillations and their possible abnormal dynamics in human amblyopia have, however, remained unknown. The variety of functional perceptual deficits as well as changes in lateral inhibition^14^ and connectivity^44^ suggests that abnormal oscillation dynamics and imprecise temporal coordination could be affected also in amblyopia. Supporting this idea, oscillation amplitudes are reduced in hemianopia^40^ where the visual deficits have an organic cause, and in animal models of strabismic amblyopia^45^. All these data together suggest that visual deficits in anisometric amblyopia could partially arise from imprecise temporal coordination in visual cortical circuits which would be reflected in abnormal cortical oscillations in human MEG and EEG.

In this study, our overarching objective was to resolve whether amblyopia is associated with reduced oscillation amplitudes. To link our study to previous work, we also investigated the correlation of reduced event-related responses with amblyopia. Our specific objectives were to (1) replicate whether event-related responses would be reduced in amblyopia using source-reconstructed MEG data, (2) localize the cortical sources of suppressed evoked activity that have remained unmapped, (3) investigate whether visual processing in amblyopia is characterized by abnormal cortical oscillations, (4) localize the cortical sources of oscillatory deficits, and (5) investigate whether reduced evoked activity and cortical oscillations would be correlated with deficits in visual acuity. To this end, we recorded MEG from 15 anisometropic amblyopic patients and from 11 healthy control subjects during visual discrimination and visual change-detection tasks which in previous studies have been associated with modulation of cortical oscillations in the visual system^36–39,46,47^. We then identified task-related modulations of event-related activity and oscillation amplitudes from source-reconstructed MEG data and compared the processing of the AE with that of the FE and, moreover, the visual processing in amblyopic patients with healthy control group. These measures were then correlated with ophthalmological measurement of visual acuity to address their functional relationship with amblyopia.

## Results

To investigate neuronal correlates of anisometric amblyopia, we estimated ERs and oscillatory activities from source-modelled MEG data recorded during two visual tasks known to be associated with modulations of oscillation amplitudes^26,28,36,46^. Stimuli were presented either to the AE or FE of adult anisometric amblyopia patients and dominant and non-dominant eye (DE and non-DE) of the control participants. To obtain a comprehensive view on electrophysiological correlates and to avoid circular hypothesis testing^48^, we used a data-driven approach, in which we estimated the difference in these measures in all cortical parcels. We first estimated the difference in ERs and oscillations amplitudes for stimuli presented to AE and FE and localized the cortical sources of these differences. To confirm that the identified differences in neuronal activity were not caused by facilitated processing in FE, we next compared the differences in these responses between patients’ FE and controls’ DE. Finally, we tested whether the reduction of visual acuity between FE and AE were correlated with neuronal activity as measured with ER and oscillation amplitudes.

### Performance with amblyopic eye is poorer than with fellow eye in T2

**Figure 1:**
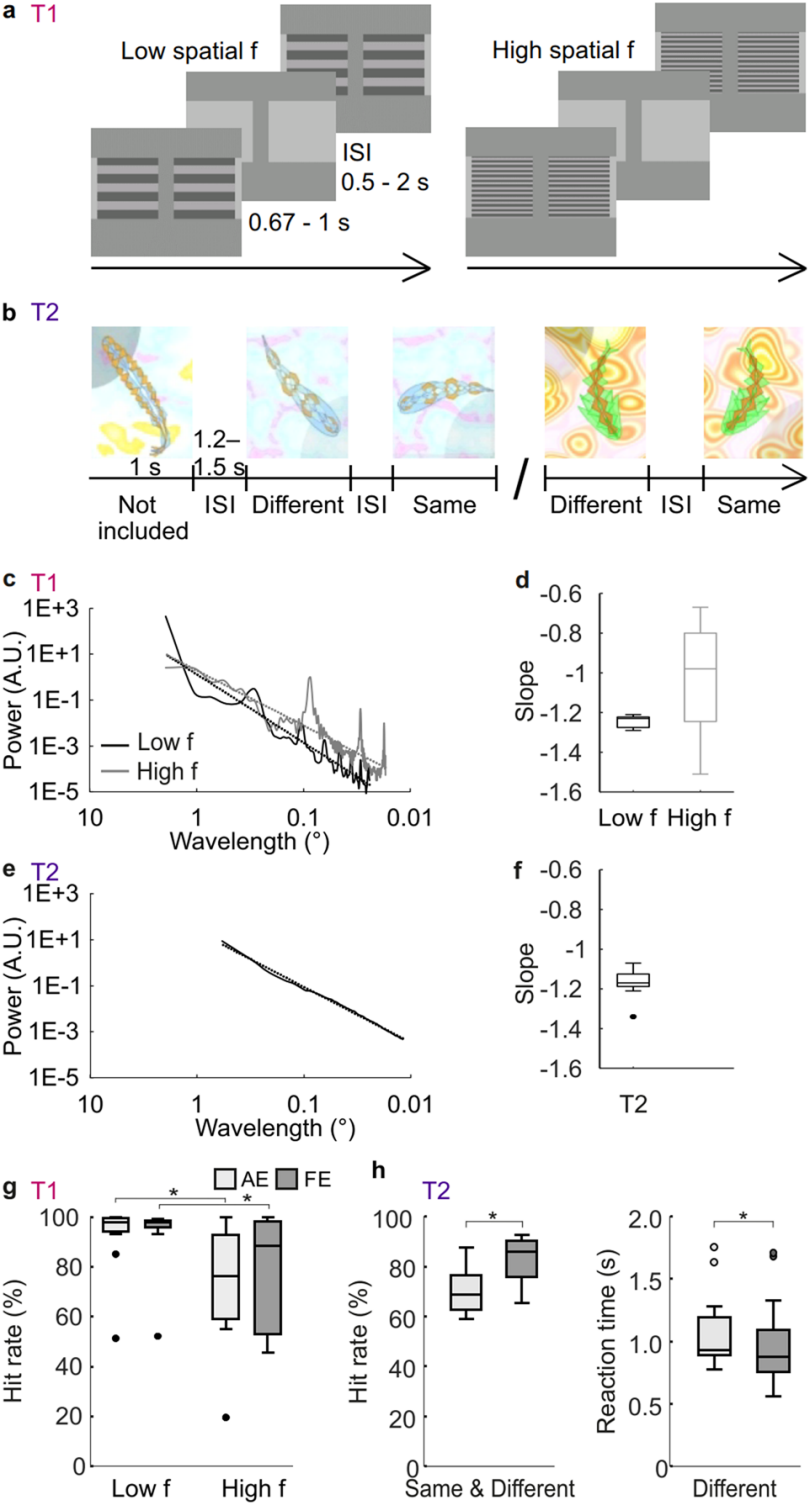
Tasks, stimuli and behavioural performance. (**a**) Schematic illustration of the discrimination task (T1) with low and high spatial frequencies. The subjects’ task was to indicate whether gratings were moving in same or different directions. (**b**) Example trials and stimulus sequences used in change-detection task (T2) shown from two different blocks with different background images. (**c**) Stimulus spectral properties as estimated with radially averaged power spectrum for T1 across stimuli views. Solid lines depict power at spatial wavelength of stimuli images, and dashed lines regression lines which are used to obtain the slopes. (**d**) Slopes of stimuli images’ power spectra for the T1 conditions as estimated with linear regression from the log–log (frequency, power) coordinates. (**e**,**f**) Same as (**c**,**d**), but for T2. (**g**) HRs for T1. Stars mark significant difference (*t*-test, *p* < 0.05). (**h**) HRs and RTs for T2. In boxplots median is marked with a line, and the whiskers extend at maximum to 1 × the interquartile range.

We first evaluated the behavioural performance in the discrimination task, T1 (Fig. 1a) and in the visual change-detection task (Fig. 1b) separately for the amblyopic eye (AE) and the fellow eye (FE) for the patient cohort. To estimate the complexity and spatial frequencies of the stimuli in the tasks, we used radially averaged power spectra and their slopes that were averaged over one example image per each task condition and per patient (Fig. 1c–f). T1 high spatial frequency condition and T2 had similar spectral slopes (*t*-test T1_high_ vs. T2: *t*(10) = 1.425, *p* = 0.185, uncorrected), while T1 had lower slopes—that is less power at high spatial wavelength (*t*-test T1_high_ vs. T1_low_: *t*(10) = 2.485, *p* = 0.032, uncorrected).

In T1, mean hit rates (HR) for the low frequency stimuli were 92.8% for the AE and 93.4% for the FE, and for the high frequency stimuli 73.8% for the AE and 75.5% for the FE (Fig. 1g). We did not find statistically significant difference in HRs for stimuli presented to the AE and the FE (*t*-test low f: *t*(10) = − 0.5138, *p* = 0.6186, high f: *t*(10) = − 0.22524, *p* = 0.8263), but found a statistically significant difference between the low and high spatial frequency versions of the task (*t*-test AE: t(10) = 2.3136, *p* = 0.04324, FE: *t*(10) = 2.591, *p* = 0.02690, uncorrected). In the visual change-detection task, T2, in which correct performance required identification of visual object features, HRs were significantly larger (*t*-test, t(12) = 4.1181, *p* = 0.001426) (Fig. 1h), and RTs were significantly faster (t(12) = − 2.4562, *p* = 0.03025) for stimuli presented to the FE than AE.

In T1, visual acuity was correlated with HRs in the high frequency condition for AE but not for FE (Spearman’s correlation test AE: *r* = − 0.6347, p = 0.03592; FE: *r* = − 0.2397, p = 0.4778). Also the difference in the visual acuity between FE and AE was correlated with the differences in HRs (FE-AE: *r* = − 0.6073, p = 0.04752). In the low frequency condition, visual acuity values were not correlated with HRs (Spearman’s correlation test AE: *r* = 0.05543, *p* = 0.8714; FE: *r* = 0.3665, *p* = 0.2677; FE-AE: *r* = − 0.06452, *p* = 0.8505). In T2, visual acuity values for AE but not for FE were correlated with HR (Spearman’s correlation test AE: *r* = − 0.5683, *p* = 0.04273; FE: *r* = 0.005548, *p* = 0.9856). Also difference in the visual acuity values between the eyes was correlated with the difference in the HRs (FE-AE: *r* = − 0.7080, *p* = 0.006772).

Next we tested whether the frequency of large eye movements (> 50 µV) differed between the trials for AE and FE. We found no significant differences in proportion of eye movements in T1 or in T2 [T1: Proportion for FE 0.224 ± 0.182 (N trials with large eye motions/N all trials, mean ± SD), for AE 0.283 ± 0.214, (FE-AE paired *t*-test *t*(10) = − 1.956, *p* = 0.0790, uncorrected); T2 Proportion for FE 0.449 ± 0.305, for AE 0.517 ± 0.288, (FE-AE paired *t*-test *t*(12) = − 1.443, *p* = 0.175, uncorrected)]. Therefore, differences in neuronal activity in the subsequent analyses cannot be explained by different amount of eye motion artifacts remaining in data after cleaning data with ICA (see “Methods”).

### Evoked activity is reduced for the AE across the visual system

**Figure 2:**
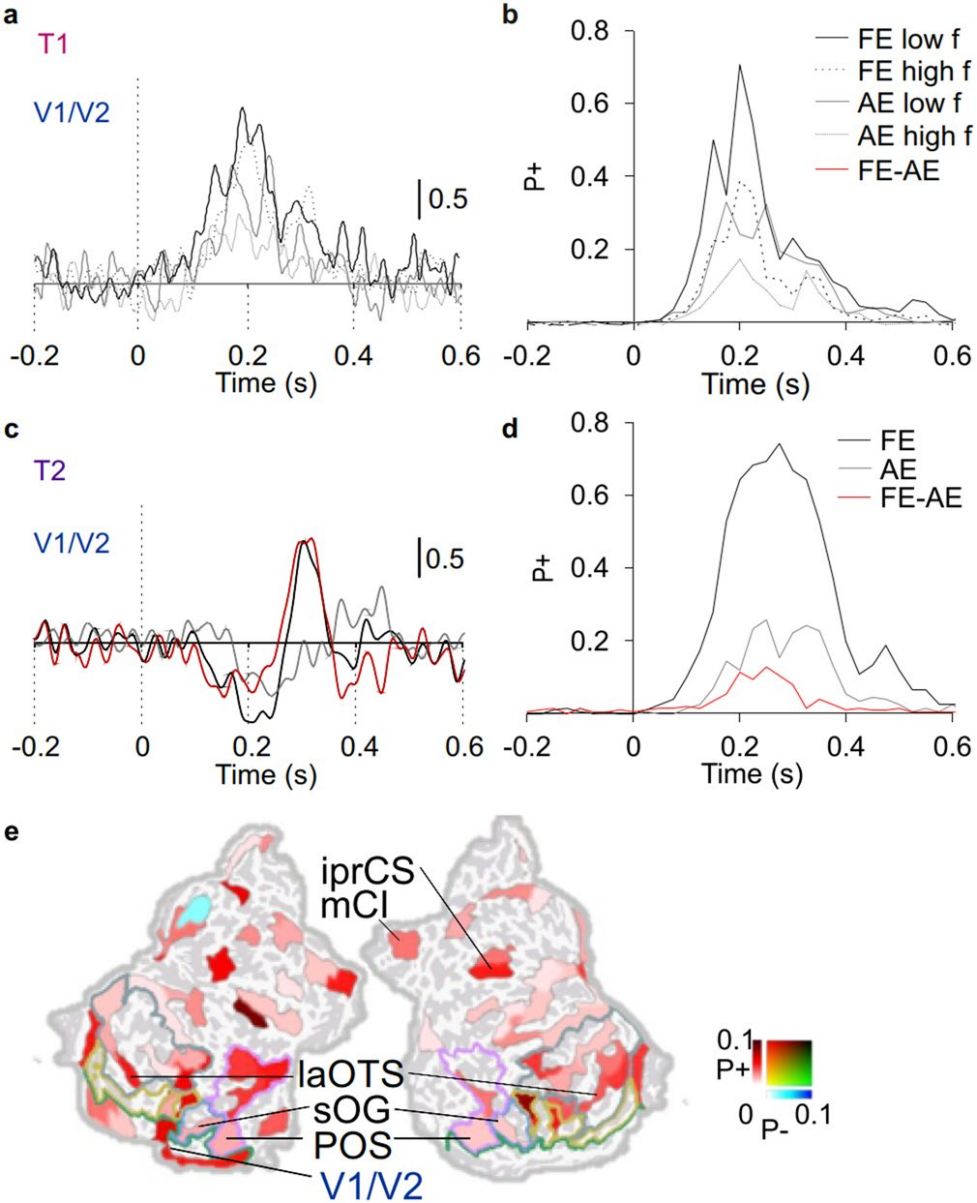
ERs are reduced for AE in T2. (**a**) ER waveforms in V1/V2 for T1. (**b**) The fraction of parcels (*P*+) showing significant ER compared to the baseline. Black line denotes fraction of parcels for FE and grey line that for AE (*t*-test, threshold *p* < 0.05, FDR corrected). (**c**,**d**) Same as in (**a**,**b**) for T2. Black line denotes the fraction of parcels for FE, grey for AE. The red line indicates fraction of parcels where the difference in the strengths of ERs between FE and AE ER was significantly different (*t*-test, threshold *p* < 0.05, FRD corrected, see “Methods”). (**e**) Cortical parcels in which ERs were stronger for stimuli presented to FE than AE in T2. Colours indicate the fraction of parcels with positive (red) or negative (blue) difference over 0.1–0.4 s. The coloured lines define boundaries for functional visual regions of interest (blue: lateral occipital, green: V1/V2, grey: ventral, pink: dorsal, yellow: V4–V8^49–51^.

Prior studies using EEG/MEG sensor level data have observed reduced evoked activity for the stimuli presented to AE compared to that of the FE^26–31,40^. To resolve whether the tasks used in the present study would be associated with reduced evoked activity in the source-level MEG data, we first investigated whether ERs would be reduced in visual discrimination task for stimuli presented to the AE compared to that of the FE and whether this suppression would be related to spatial frequency of the stimuli. We visualized the ER waveforms over primary visual cortex (V1/V2) but found no significant differences between the FE and the AE (Fig. 2a, *t*-test, threshold *p* < 0.05, FDR corrected). We then estimated differences in the absolute-valued ERs compared to pre-stimulus baseline separately for the FE and the AE and for each cortical parcel (*t*-test, threshold *p* < 0.05, FDR corrected). These data were then represented as the number of parcels, in which ERs showed significant modulation (Fig. 2b) as well as the mean strength averaged over parcels with significant difference (Supplementary Fig. 1). Both low- and high-frequency stimuli induced clear ERs both when stimuli were presented to the AE and FE in the primary and secondary visual cortices V1/V2 (Fig. 2a), but the number of significantly activated parcels (Fig. 2b) and strength (Supplementary Fig. 1a) were smaller when stimuli were presented for the AE. We then estimated whether the ERs were statistically significantly different between those stimuli presented to the AE and FE, but there were no differences in the strength of ERs between stimuli presented to the FE and AE in any of the parcels (Fig. 2b).

We next visualized the ER waveforms over V1/V2 for T2. The initial deflection and a subsequent increase at 150–300 ms from stimulus onset characterized ER in the V1/V2 for the FE but not for the AE, these differences being significant (Fig. 2c, *t*-test, threshold *p* < 0.05, FDR corrected). We then estimated separately for each cortical parcel ERs for the FE and AE in T2 as described above for T1. ERs for stimuli presented to the FE were considerably more robust being significant in 74% of parcels (Fig. 2d), as well as stronger (Supplementary Fig. 1b) than those presented to the AE which activated only 26% of the parcels (*t*-test, threshold *p* < 0.05, FDR corrected). In line, ERs were also stronger for the FE than AE, this difference peaking at 250 ms from stimulus onset in 13% of the parcels (Fig. 2d, *t*-test, threshold *p* < 0.05, FDR corrected).

To resolve the cortical regions in which ERs differed between stimuli presented to AE and FE in T2, we next visualized the cortical origins of reduced ERs. To facilitate functional interpretations of these data, we also delineated early visual (V1–V2), lateral occipital cortex (LOC), V4–V8, as well as ventral and dorsal parts of the visual regions^49–51^ (see “Methods”). ERs were reduced for AE compared to FE in few parcels of V1/V2, LOC, V4–V8, as well as in lateral occipito-temporal cortex (laOTC) of the ventral stream and in parieto-occipital sulcus (POS) of the dorsal stream (Fig. 2e).

### AE is associated with reduced stimulus induced oscillation amplitude modulations compared to the FE

**Figure 3:**
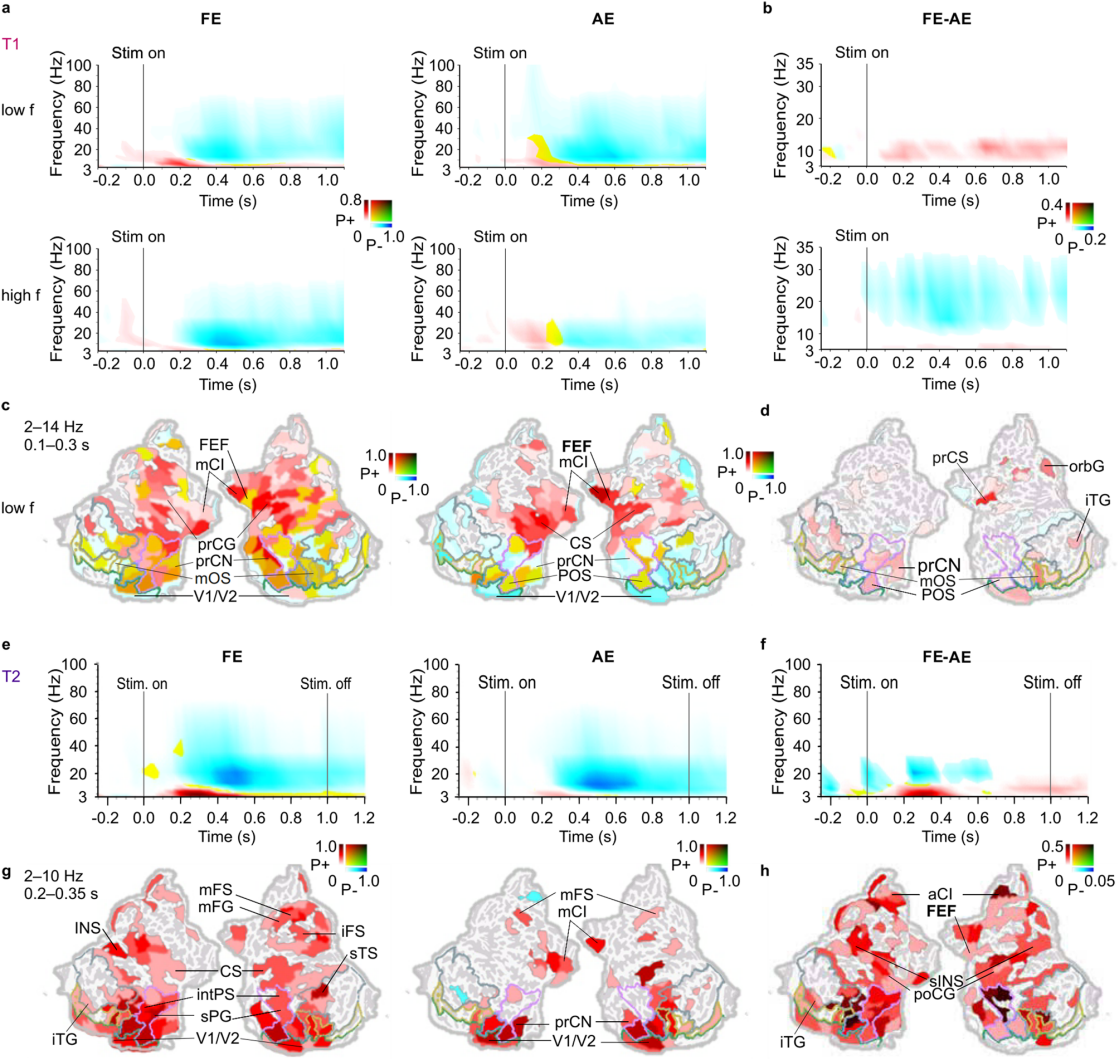
Oscillation amplitudes are stronger for FE than AE. (**a**) Time–frequency representations (TFR) for spectrally resolved oscillation amplitude modulations separately for stimuli presented to the FE and AE compared to baseline (*t*-test, threshold *p* < 0.05, FDR corrected) for T1. Colour codes show the fraction of parcels with statistically significant positive (P+, red) or negative (P−, blue) modulation or their mixture (yellow/green) and the coloured lines define boundaries for functional visual regions of interest as in Fig. 2. (**b**) TFRs for the difference in oscillation amplitudes between FE and AE (*t*-test, threshold *p* < 0.05, FDR corrected). (**c**) Cortical sources for oscillation amplitudes averaged over time and frequency selections indicated in A for FE (*t*-test, threshold *p* < 0.01, FDR corrected) and for AE (*t*-test, threshold *p* < 0.05, FDR corrected). Colours indicate the strength of the amplitude modulation for positive (P+, red), negative (P−, blue) difference as well as the presence of both (yellow/green). (**d**) Cortical sources showing differences in oscillation amplitudes between FE and AE (*t*-test, threshold *p* < 0.05, FDR corrected) averaged over time and frequency selections indicated in (**b**). (**e–h**) Same as (**a**–**d**) but for the T2. *A* anterior, *la* lateral, *m* middle, *i* inferior, *int* intra, *orb* orbital, *po* post, *pr* pre, *s* superior, *CI* cingulate, *CN* cuneus, *FEF* frontal eye field (posterior part of superior prefrontal sulcus), *INS* insula, *C* central, *F* frontal, *P* parietal, *T* temporal, *O* occipital, *G* gyrus, *S* sulcus.

We next evaluated if also oscillation amplitude modulations, that are typically associated with the processing of visual information^34–38^, were suppressed for stimuli presented to the AE. We computed stimulus induced oscillation amplitude modulations for stimuli presented to AE and FE separately for each parcel and for each frequency. We then visualized these data as time–frequency representations (TFR), where we represented the fractions of cortical parcels with statistically significant amplitude changes from the baseline level as a function of time and frequency (*t*-test, threshold *p* < 0.05, FDR corrected). In T1, both low- and high-frequency stimuli were associated with classical pattern of an early theta-alpha (2–14 Hz) band amplitude increase and subsequent wide band (4–70 Hz) amplitude suppression both when stimuli presented to the FE and to the AE (Fig. 3a). The strength of oscillations amplitudes differed between stimuli presented to the FE and AE for both low frequency and high frequency stimuli. For the low-spatial-frequency stimuli, both early transient and sustained theta-alpha amplitudes were larger when stimuli were presented to the FE compared to the AE, while for high-spatial-frequency stimuli, the alpha–beta band suppression was larger for the stimuli presented to the FE (*t*-test, threshold *p* < 0.05, FRD corrected) (Fig. 3b). The amplitude difference between FE and AE was larger for low- than high-spatial frequency stimuli (Supplementary Fig. 2). Notably, there were no observable differences in the oscillation amplitudes at the temporal frequency of the grating (3.2 Hz) indicating that differences in oscillatory activity between AE and FE were not driven simply by brain responses to temporal frequency. Next to resolve the cortical origin of oscillation amplitudes for FE and AE, we visualized the significant effects in cortical anatomy. In the visual discrimination task, theta–alpha-band amplitudes for stimuli presented to the FE were associated with induced oscillations in dorsal and ventral visual stream regions as well as in premotor areas including frontal eye fields (FEF) while for AE oscillations were localized to primary visual and premotor regions (Fig. 3c). Oscillations were stronger for the FE than to the AE in primary visual areas V1/V2, in LOC and inferotemporal gyrus of the temporal cortex i.e. across ventral and dorsal visual stream regions (Fig. 3d).

The change-detection task, T2, also evoked a classical spectro-temporal pattern of an early theta–alpha (2–10 Hz) band amplitude increase and subsequent wide band (4–40 Hz) amplitude suppression compared to baseline both when presented to the FE and the AE (Fig. 3e) (*t*-test, threshold *p* < 0.05, corrected). Theta–alpha band amplitudes were also larger when stimuli were presented to the FE in comparison to the AE (*t*-test, threshold *p* < 0.05, corrected) (Fig. 3f). For stimuli presented to the FE, this transient amplitude increase originated from nearly all parcels of the visual ventral and dorsal stream regions and extended to higher order visual areas as well as to intraparietal sulcus (intPS) in the posterior parietal cortex (PPC) and middle frontal gyrus/sulcus (mFG/mFS) in the lateral prefrontal cortex (lPFC) (Fig. 3g). When stimuli were viewed with the AE, the amplitude increase was observed in V1/V2, but it did not extend to higher order ventral stream or dorsal stream regions. The amplitudes also were larger for the FE than for the AE in nearly all parcels of the visual systems as well as in superior precentral sulcus corresponding functionally to frontal eye fields (FEF), intPS, and several parcels of the lPFC including mFG/mFS (Fig. 3h).

### Differences in the event-related responses between amblyopic patients and control participants

**Figure 4:**
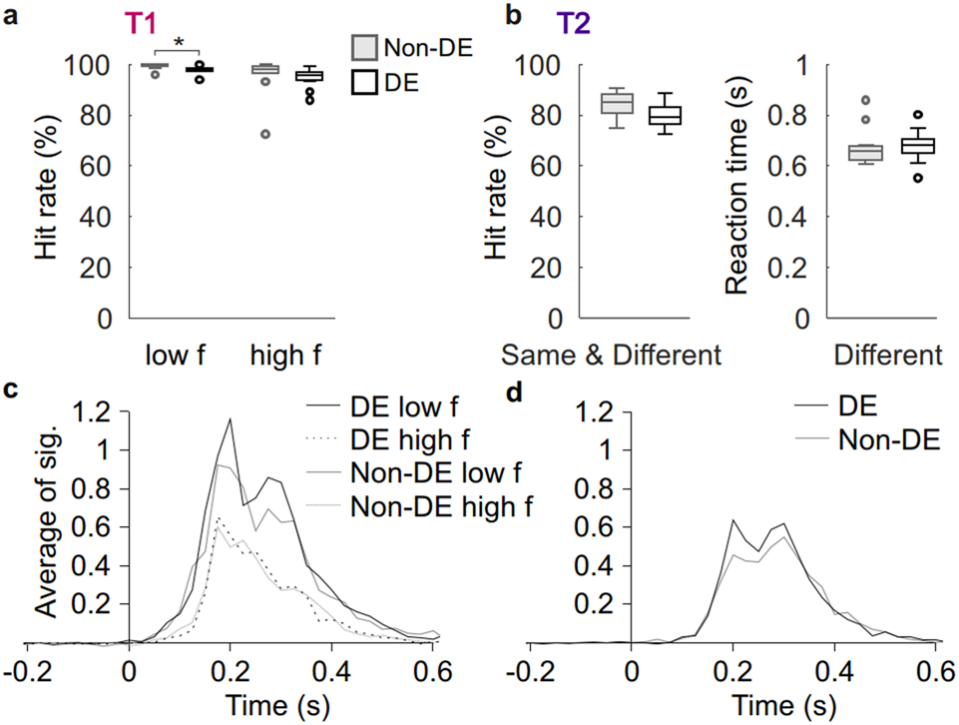
Performance and ERs for control participants. (**a**) HRs for the DE and Non-DE in T1. Stars mark significant difference (*t*-test, *p* < 0.05). (**b**) HRs and RTs in T2. Note that if the stimuli is “Same”, the participant is to withhold using the response pad. (**c**) ERs in T1 and (**d**) in T2. Axes and scales as in Fig. 2. In boxplots median is marked with a line, and the whiskers extend at maximum to 1 × the interquartile range.

In order to test the notion that the processing of the FE could be facilitated due to lack of feedback from the AE^14,26,27^, we next investigated if not only the processing of the AE but also the processing of the FE would be altered compared to healthy participants. We first confirmed that there were no or only small differences in HRs between control subjects’ DE and non-DE. In the discrimination task HRs were slightly different in the low frequency condition (mean of the difference 1.34% smaller for non-DE), and similar in the high frequency condition (Fig. 4a, *t*-test, Low frequency: t(10) = − 2.8571, *p* = 0.01704; High frequency: t(10) = − 0.42817, *p* = 0.6776). In the change-detection task, HRs and RTs were not significantly different for the DE and the non-DE (Fig. 4b, HR, t(10) = − 2.1642 *p* = 0.05571; RT t(10) = 0.19203, *p* = 0.8516). In T1, visual acuity values were not significantly correlated with HRs in neither of the conditions (Spearman’s correlation test low frequency condition non-DE: *r* = 0.03949, *p* = 0.9082; DE: *r* = − 0.1957, *p* = 0.5641; DE-non-DE: *r* = − 0.2651, *p* = 0.4308; high frequency condition non-DE: *r* = 0.2512, *p* = 0.4563; DE: *r* = − 0.3920, *p* = 0.2331; DE-non-DE: *r* = 0.5254, *p* = 0.09699). In T2, better acuity of DE was correlated with better HR (non-DE: *r* = 0.009175, *p* = 0.9786; DE: *r* = − 0.6776, *p* = 0.02195; DE-non-DE: *r* = − 0.1150, *p* = 0.7365).

ERs were then computed separately for stimuli presented to the DE and the non-DE in the discrimination, T1, (Fig. 4c) and change detection, T2, (Fig. 4d) tasks. Similar patterns of ERs were observed in all conditions, the difference between the DE and the non-DE being non-significant (*t*-test, *p* > 0.05). Similarly, also oscillation amplitudes reproduced neuronal patterns observed for the FE and were largely similar between the DE and the non-DE in both tasks (Supplementary Fig. 3).

**Figure 5:**
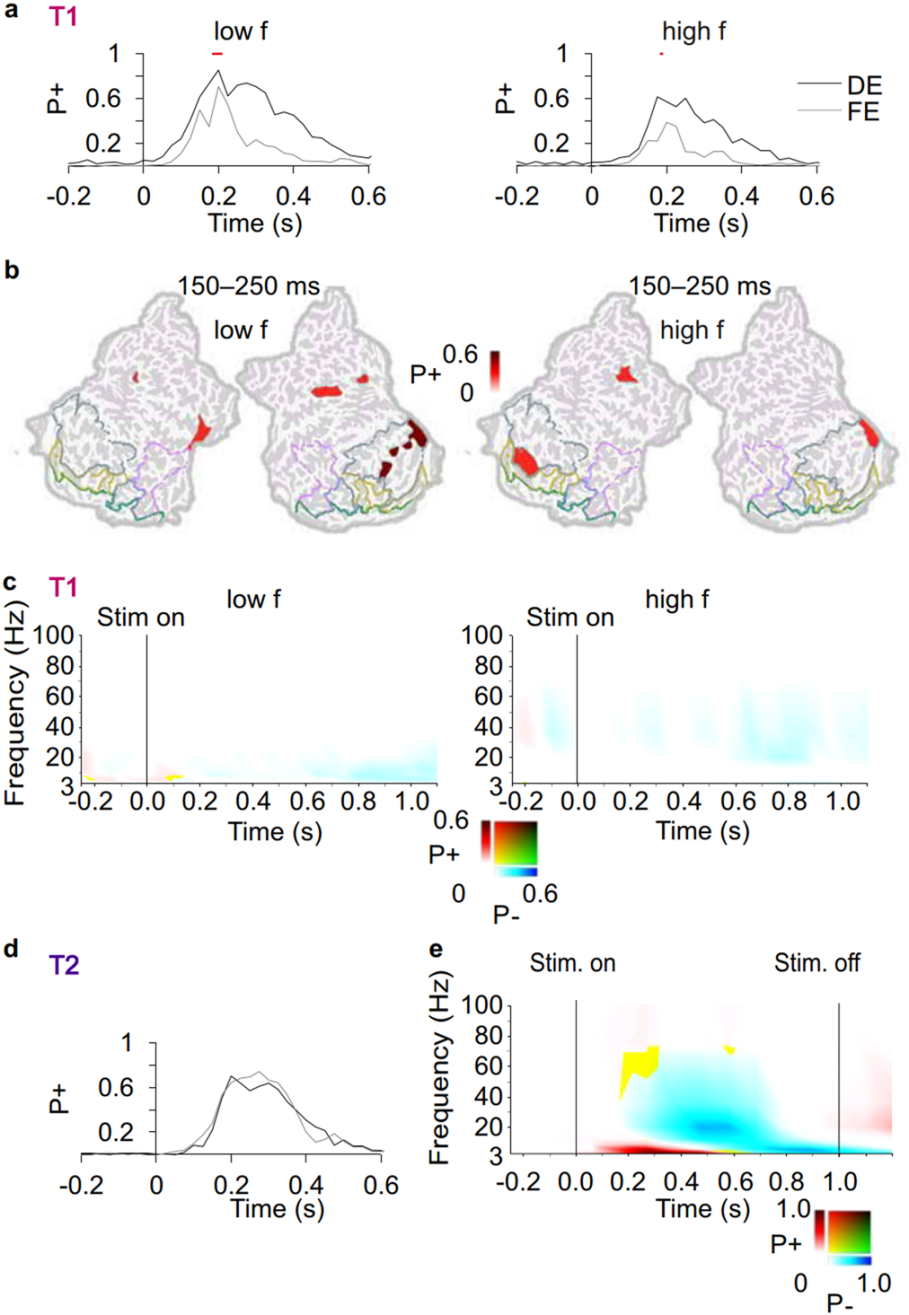
Neuronal processing is not facilitated for the FE compared to control subjects. (**a**) The fraction of parcels in which ER showed significant modulation compared to baseline for DE of the control subjects and the FE of the patients (*t-*test, *p* < 0.05, FDR corrected) in T1. The red lines indicate statistical differences between DE and FE (Welch’s *t*-test, *p* < 0.05, FDR corrected). (**b**) Cortical locations of the ER differences between DE and FE. (**c**) Oscillation amplitude differences between DE and FE in T1 (Welch’s *t*-test, *p* < 0.05, FDR corrected). (**d**) Same as a but for T2. (**e**) Same as c but for T2.

We then compared how the processing of visual information differed between the patients’ FE and control participants’ DE. In the discrimination task T1, HRs were significantly greater for the DE compared to the FE for high-spatial frequency stimuli (Welch’s *t*-test, high frequency t(10.577) = − 2.6877, *p* = 0.02178; low frequency t(10.241) = − 1.0832, *p* = 0.3036). In the change-detection task T2, RTs were lower for the DE than for the FE (Welch’s *t*-test, t(12.938) = − 3.0398, *p* = 0.00953) but there was no difference in HRs (t(13.906) = 1.2132, *p* = 0.2453). Thus in contrast with the hypothesized superiority of FE over DE, we found behavioural evidence for the superiority of DE over FE in both tasks. In the discrimination task, the FE and the DE differed for a very brief duration in ER peak strength at 200 ms (Welch’s *t*-test, *p* < 0.05, corrected, Fig. 5a,b). Also, the low frequency amplitude modulations were slightly stronger for the DE than for the FE (Welch’s *t*-test, *p* < 0.05, corrected) (Fig. 5c). In the change-detection task, the strength of ERs between the FE and the DE did not differ (Fig. 5d), but oscillation amplitude changes were more pronounced for the DE than FE (Fig. 5e, see also Supplementary Fig. 3). We further tested if there were different proportions of large eye motions (> 50 µV) in trials between FE and DE. We found no significant differences in T1 or T2 (T1 FE 0.283 ± 0.214 (N trials with large eye motions/N all trials, mean ± SD), for DE 0.141 ± 0.156 (FE-DE Welch Two Sample *t*-test *t*(19.549) = 1.151, *p* = 0.264, uncorrected); T2 for FE 0.449 ± 0.305, for DE 0.338 ± 0.265 (FE-DE Welch Two Sample *t*-test t(21.977) = 0.955, *p* = 0.350, uncorrected).

### Oscillatory responses are correlated with visual acuity

**Figure 6:**
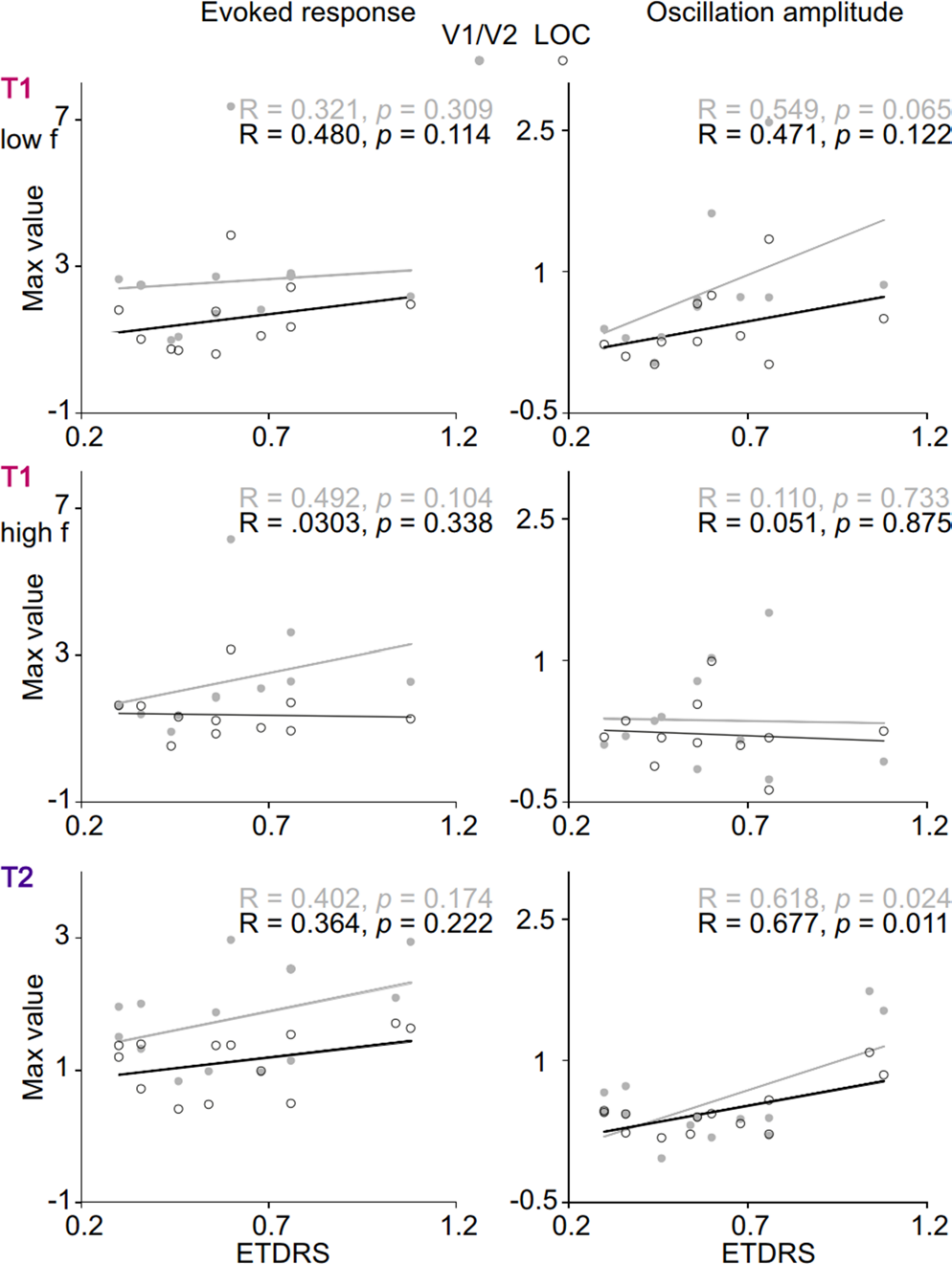
Oscillation amplitudes correlate with depth of amblyopia in the change detection task. Correlation of ERs and oscillation amplitudes with the depth of amblyopia (Pearson’s correlation tests, Bonferroni corrected with 6 tests) as indexed by the visual acuity of the AE (ETDRS values). The correlation was based on maximum peak values of the FE-AE difference in the ERs and theta-alpha oscillations between 0.1 and 0.4 s. Filled grey points denote V1/V2 parcels selection, and open black points lateral occipital cortex (LOC) parcels selection.

Finally, we addressed whether visual acuity deficits in amblyopia could be correlated with reduced oscillation amplitudes and ERs. We selected the maximum values of FE-AE differences in ERs and oscillation amplitudes over the 0.1–0.4 s and averaged over parcels in V1/V2, and those of LOC and V4–V8 (see “Methods”). We then estimated whether these differences in neuronal activity were correlated in the reduction of the visual acuity between FE and AE as estimated with LogMAR values. Reduction in the ERs in neither V1/V2 or LOC were correlated with the reduction in the visual acuity in the discrimination task in the low frequency condition (Pearson’s correlation test, V1/V2, *r* = 0.321, *p* = 0.309; LOC, *r* = 0.480, *p* = 0.114), high frequency condition (V1/V2, *r* = 0.492, *p* = 0.104; LOC, *r* = 0.0303, *p* = 0.338) or change-detection task (V1/V1, *r* = 0.402, *p* = 0.174; LOC, *r* = 0.364, *p* = 0.222) (Fig. 6). In contrast, reduction of oscillation amplitudes was correlated with the reduction of visual acuity in the change-detection task both in V1/V2 and LOC (V1/V2, *p* = 0.024, *r* = 0.618; LOC, *p* = 0.011, *r* = 0.677) but not in the discrimination task low frequency condition (V1/V2, *r* = 0.549, *p* = 0.065; LOC, *r* = 0.471, *p* = 0.122) or high frequency condition (V1/V2, *r* = 0.110, *p* = 0.733; LOC, *r* = 0.051, *p* = 0.875). Finally, we tested whether the correlation of amplitudes with visual acuity was specific to visual deficits in amblyopia or whether it would also be correlated with visual acuity in healthy participants. Considering the small differences in visual acuity in controls, unsurprisingly, we did not observe any correlation between visual acuity and neuronal activity in the healthy control group (ER and LogMAR correlation range in both tasks and V1/V2 and LOC: − 0.282 < *r* < 0.356, most significant *p* = 0.279, uncorrected; Oscillation amplitudes correlation range in both tasks and V1/V2 and LOC: − 0.109 < *r* < 0.444, most significant *p* = 0.171, uncorrected).

## Discussion

An understanding of the neuronal basis of brain diseases at different scales of research supports the development of new therapeutic approaches. However, while there are several lines of research on the neuronal underpinnings of amblyopia at the cellular and microcircuit levels, understanding of the systems-level neuronal deficits contributing to amblyopia has remained limited. Abnormal oscillations and synchronization are thought to contribute to symptoms and emergence of many neurodevelopmental brain diseases^23,41,42,52^, oscillations being attenuated in hemianopia^40^ and in animal models of strabismic amblyopia^45^. We tested here the hypothesis that visual ERs and oscillation amplitudes would be reduced in anisometric amblyopia and that these reductions would be correlated with decreased visual acuity, suggesting a relationship between the systems-level neuronal dynamics and the perceptual deficit. Using source-constructed MEG data together with visual-discrimination and change-detection tasks, we found here that ERs and low-frequency oscillation amplitudes were indeed reduced when visual stimuli were presented to the amblyopic eye (AE) compared to the fellow eye (FE) in anisometric amblyopic patients (see Supplementary Table 1). For the AE, event-related neuronal processing in early visual regions was qualitatively similar to, albeit quantitatively weaker, than for FE. However, for AE stimuli, the oscillation-amplitude responses reached neither the higher-level visual regions nor the parietal and prefrontal brain regions. Overall, to the best of our knowledge, our study is the firsts to reveal neuroanatomically limited cortical oscillations in anisometric amblyopia and the cortical sources of reduced ERs. Critically, we found that the attenuated oscillation-amplitude responses for AE stimuli, but not the ERs, were correlated with the decreased visual acuity of the AE, suggesting that impaired neuronal oscillations in large-scale cortical circuits play a role in the genesis of the visual perceptual deficits in anisometric amblyopia. These findings are in line with the roles attributed to neuronal oscillations in supporting adult perceptual functions^32,33^. Importantly, oscillations and synchronized neuronal activity play a crucial role in the experience-dependent development of cortical networks^53,54^. Thereby, abnormal cortical oscillations in adult amblyopia may arise from abnormal visual experiences in childhood impacting synchronization of neuronal activity and leading to plastic modifications in development of visual networks. Novel therapies aiming to restore vision in AE could putatively be targeted on these oscillatory dynamics arising from interactions and circuit dynamics of pyramidal neurons and inhibitory neurons (INs), especially those expressing parvalbumin^42,55^.

Behavioural performance was decreased for stimuli presented to the AE in a change-detection task where the correct identification of visual stimuli demanded accurate identification of visual details. These data are in line with prior studies indicating that visual acuity in amblyopia is compromised specifically for stimuli with high spatial frequencies^14,56–58^ and for low contrast^5,6^. In our data for the change-detection task, RTs for stimuli presented to patients’ FE were increased compared to control participants’ DE, showing that behavioural deficits in amblyopia also extend to the FE. These data support the view in which amblyopia may be partially caused by deficits in lateral inhibition between the eyes^13,44,59,60^. In T1, in which the task was to detect direction of motion of two moving gratings, the high spatial frequency was calibrated prior to the onset of MEG recordings to be barely visible. In line with this procedure, the performance was similar when viewed with FE and AE.

Albeit many prior studies have shown that ERs are attenuated for stimuli presented to the AE compared to the FE in EEG scalp potentials^26,27,30,31^, only few prior studies have found such evidence in MEG sensor fields^28,29^ and the cortical sources of the attenuated ERs have remained largely unidentified. ERs for the AE were attenuated in the change-detection task wherein the performance depended on the accurate representation of visual details, but not in the discrimination task. ERs were reduced in primary visual areas but also along the ventral visual stream which supports object recognition^51,61^. These results are in line with an fMRI-guided EEG study showing that attentional modulations in ERs are reduced for stimuli presented to the AE in both early and late visual regions^30^. One should note, however, that in our study, ERs were estimated separately for each eye, the other being occluded. Occlusion has been proposed to have an impact on stimulus detection and perception so that after deprivation, gratings viewed by the deprived eye appeared of higher contrast than those viewed by the non-deprived eye, but detection thresholds remain virtually unaffected^62^. However even with this possible effect, we found greatly reduced responses to stimuli presented to AE. In our experiments, occlusion lasted for 12–15 min at a time, which should not have a noticeable impact on visual processing.

In both tasks, oscillation amplitudes showed a conventional pattern of an early transient low-frequency theta-alpha band amplitude increase and subsequent sustained wideband suppression for the stimuli presented to the FE. Similar observations have been reported in a number of prior studies to characterize both visual perception^34–38,63,64^ and change-detection tasks^36,46,65^.

In the discrimination task (T1), the amplitudes were reduced for the AE in the ventral stream regions but also in dorsal visual stream areas such as POS and precuneus, both of which have been linked to motion perception in prior fMRI studies of healthy participants^66,67^. In the change-detection task, reduced theta–alpha oscillation amplitudes for the AE were observed in primary visual regions ventral visual stream regions processing of object features and identity^51,61^ and in which prior MEG studies have found oscillation amplitudes to be correlated with visual perception and change-detection tasks^36–38,46^. The lack of activation of higher-order visual regions, specifically prominent in the change-detection task, suggests that the perceptual deficits in amblyopia may stem from limited functional activation of the visual system and inadequate oscillatory activity therein. In addition to the modulation of oscillation amplitude in visual system, oscillations were reduced also in PPC and PFC which implies that that PPC and PFC become dysfunctional when visual inputs from the AE are inadequate. Apparently, only one prior study has analysed oscillatory amplitude modulations for stimuli presented to the AE using EEG sensor level analysis but found no differences in these measures^31^. The differential results compared to our study could be due to different paradigms but more likely reflect the greater sensitivity of MEG source level analysis. Future studies are needed to systematically investigate whether reduced oscillation amplitudes are a general feature of amblyopic visual processing or limited to processing of certain kinds of visual stimuli and behavioural tasks.

Amblyopia is thought to be caused by abnormal neuronal suppression between the eyes^13,44,59,60^ and by excessive suppression from the FE to the AE^20^ that is specifically pronounced for high spatial frequencies^14^. This is evidenced by both behavioural^6,60^ and repetitive transcranial magnetic stimulation (rTMS)^59^ studies which have shown that inter-ocular balance is also altered. Therefore, although the FE has a normal visual acuity, these studies have suggested that neuronal processing could also be facilitated for the stimuli presented to the FE because of the lack of feedback inhibition from the AE. Hence, suppressed neuronal activity for stimuli presented to the AE compared to the FE could also be due to increased processing for the FE rather than to reduced processing for the AE. Yet, both the time–frequency and anatomical patterns of ERs and oscillation amplitudes obtained from control subjects with normal visual acuity reproduced largely the observations obtained from the FE of anisometric amblyopic patients. Furthermore, neuronal activity was more strongly modulated by stimuli shown to the DE of healthy participants than to the FE of amblyopic patients. This indicated that stimuli presented to the FE were not associated with facilitated neuronal processing but rather that also the processing of these stimuli was task-dependently weaker or equal compared to healthy visual system. Hence, facilitated neuronal processing for FE cannot explain the observed FE-AE differences in our study, which therefore indicate that stimuli presented to AE are associated with reduced neuronal processing in the form of ERs and oscillation amplitudes.

To investigate whether reduced oscillation amplitude dynamics as well as ERs could be predictive of visual acuity deficits in amblyopia, we correlated their strength with difference in the visual acuity between AE and FE. Importantly, strength reduction of theta–alpha band amplitudes but not that of ERs predicted strength of visual acuity. Evoked responses have been suggested to reflect neural activity evoked by the stimulus, but some studies also suggest that spontaneous or “background” neural activity that can phase-lock to the stimulus onset would contribute to evoked activity^68,69^. The different influence of amblyopia on ERs and oscillation amplitudes imply that in the present study, these phenomena reflect separate neuronal activities. Interestingly, ERs have been used as a proxy of visual acuity for instance in rodents^70^. Our data suggest that reduction of oscillation amplitudes may have a closer relationship to visual acuity deficits than ERs. Overall, the correlation of reduced amplitudes and visual acuity deficits, provide correlative evidence for that strength of oscillation amplitude dynamics may contribute to functional systems level mechanism underlying visual processing deficits in anisometric amblyopia.

## Materials and methods

### Participants and clinical eye examination

Adult amblyopic patients were recruited from a clinical second phase IIa trial (EudraCT number 2010-023216-14) investigating the effect of combined videogame and fluoxetine or placebo treatment intervention on the recovery of vision in the AE^18^. In the trial, patient diagnosed with amblyopia due to myopic or hyperopic anisometropia or congenital esotropia were included. Amblyopia was considered to be anisometropic if both refractive error was at least 1 D of anisometropia (spherical equivalent) and no strabismus had been diagnosed in childhood. Fifteen of this populations volunteered to participate in the MEG sub-study (age 43 ± 8 years, mean ± standard deviation (SD), 6 females, right-handed) (see Supplementary Table 2). All data used in this article are obtained prior to the intervention period.

Briefly, best corrected visual acuity was assessed using a large-format standardized ETDRS (charts R, 1 and 2) light box (ESV3000 with LED lights, VectorVision, Greenville, OH) placed 4 m from the subject (constant light level of 85 cd/m^2^) first in the AE as the number of correctly identified letters. Amblyopia was confirmed as an interocular ETDRS best-corrected visual acuity difference of at least two lines and/or logMAR visual acuity inclusion criteria being: amblyopic eye ≥ 0.30 and < 1.10 logMAR, dominant eye ≤ 0.10 logMAR, and anisometropia ≤ 4.25 diopters (spherical equivalent). Binocularity was examined using the Bagolini striated glass test before monocular testing^71^. Lens striations were placed at 135° before the right eye and 45° before the left eye using lorgnette frames.

Eleven healthy control subjects (age 33 ± 8 years, 3 females, right-handed) with no prior diagnosis with amblyopia were recruited. Binocularity and the best corrected visual acuity along with subjective refraction were tested as described above and spectacles were prescribed if necessary. The largest accepted interocular difference in refraction between the eyes was > 0.1 log units (one line). Eye dominance for healthy subjects was determined with hole-in-the-card test.

This study was approved by the ethical committee of Helsinki University Central Hospital (HUCH) and Finnish Medicines Agency (FIMEA) and was performed according to the Declaration of Helsinki. Written informed consent was obtained from each patient and subject prior to the experiment. The number of patients in this study was limited by the fairly small populations of Finland and Estonia and the resulting pool of amblyopia patients that fulfilled the inclusion criteria and were willing to participate to the study.

### Recordings

The MEG data were recorded with 204 planar gradiometers and 102 magnetometers (Elekta Neuromag Ltd, Finland) with sampling rate of 600 Hz. T1-weighted structural magnetic resonance images (MRI) obtained with the MP-RAGE sequence (Siemens, Germany) at ≤ 1 × 1 × 1 mm voxel (maximum voxel centre distance 1 mm, device’s average 0.98 mm) size from each participant were used for the generation of individual cortical source models for MEG source reconstruction.

### Tasks and stimuli

MEG was collected during two tasks in which stimuli were viewed with one eye the other being occluded by an opaque eye patch. Patients performed tasks first with FE to familiarize with the task, and control subjects with DE. In MEG, refraction error was corrected with contact lenses or with MEG compatible goggles. All participants responded with right thumb and/or index finger using a response pad tracking optically when fingers were lifted. The viewing distance of 16° × 12.8° display was 190 cm in both tasks.

#### Task 1 (T1): Discrimination task

We used a visual discrimination task based on grating stimuli that have been shown to induce strong oscillatory responses^72^ as well as to be associated with processing deficits in amblyopia^26,28^. Two horizontally moving gratings of 3.5° × 3.5° in the centre were shown (Fig. 1a). The gratings moved at a speed of 2.8°/s and had either a low spatial frequency (low f) of 1.14 cycles per degree (cpd) or a high spatial frequency (high f) calibrated to perceptual threshold of AE (mean ± SD, 12 ± 9.43 cpd). The duty cycle reflecting local luminance changes for the low-frequency stimuli was hence at 3.2 Hz but for the high-frequency stimuli the frequency duty cycle varied across participants. Duration of the stimuli were 670–1000 ms and inter stimulus interval (ISI) 500–2000 ms. Participants were instructed to indicate the whether the gratings moved in the same or opposite directions after the offset of stimuli. The stimulus period that was free of motor-response related activity was used for further data-analysis.

#### Task 2 (T2): Change-detection task

The visual change detections tasks have been used to induce oscillatory responses and provide a means to assess sustained neuronal activity without confounding motor responses^36,46^. Stimuli were designed to impose attention on visual details demanding visual acuity and contrast sensitivity. The task was also used as a part of the computerized visual-acuity training in the clinical trial^18^. For details see Ref.^18^ and Supplementary Materials and Methods. Briefly, to avoid influence of long-term memory and semantic associations that could be associated with common objects, complex visual objects (Fig. 1b) were designed manually prior to the experiment by a bank of additive and multiplicative simple algebraic functions. The stimuli were 1.9° ± 0.20° in size and presented in quadrants with their mid-point 1.5° from the centre fixation point of the 16° × 12.8° display. These visual objects were presented sequentially to the participants at the duration of 1000 ms ± 100 ms with ISI of 1200–1500 ms. The participants compared each object with the previous one and indicated with a response pad when its shape was different from the prior stimulus regardless of its position, size, rotation, or curvature (Fig. 1b). When the object was the same as the prior object (50% probability), the participants were to withhold responding. Only these trials were used for subsequent data-analysis to avoid any motor-response related activity. Five stimulus blocks with total of 200 stimuli were collected per eye.

To estimate the complexity and spatial frequencies of the stimuli in the tasks, we used radially averaged power spectra (RAPS) and their slopes that were averaged over one example image per each task condition and per patient. RAPS was used to obtain the average of direction-independent power spectra of the stimuli images and to visualize the 2D power spectra in 1D. The slopes were obtained from these 1D spectra with linear regression in log–log (frequency, power) coordinates.

#### Resting-state

Five minutes of eyes-open resting state was recorded after the task-blocks.

### Behavioural performance

We calculated hit rates (HR) as a proportion of trials with correct responses from all trials. Reaction times (RT) were estimated in T2 from the stimulus onset to the onset of the participant’s correct responses. In T1, responses were given after the offset of the stimuli and RTs provide no meaningful information and are not provided. Statistical significances in HRs and RTs between used eyes were evaluated with paired *t*-tests. The correlation of visual acuity (LogMAR) and task performance was estimated with Spearman rank correlation.

### Estimating the frequency of large eye movements

Large events > 50 µV reflecting blinks or large saccades were identified from electro-oculography (EOG) data recorded concurrently with MEG. The number of trials with large EOG events were identified separately for all conditions and for the stimuli presented to the FE and AE of the patient and DE of the control groups. Differences in the proportion of trials contaminated by the large EOG events out of all trials between stimuli presented to the FE and AE was estimated with paired *t*-test and between FE of patients and DE of controls with a Welch Two Sample *t*-test.

### MEG pre-processing and source analysis

Temporal extension of the signal space separation method (tSSS) was used to remove extra cranial noise from raw MEG data and independent components analysis (ICA^73^) to identify and exclude eye movement, blink, and cardiac artifacts. FreeSurfer software (http://surfer.nmr.mgh.harvard.edu/; RRID:nif-0000-00304) was used for volumetric separation of brain surfaces from MRI images, cortex flattening and splitting, and labelling cortical areas according to FreeSurfer Dextrieux atlas. Boundary element modelling (BEM), MEG-MRI co-localization, cortically constrained source modelling, and preparation of forward and inverse models was done using MNE software (http://www.nmr.mgh.harvard.edu/martinos/userInfo/data/sofMNE.php)^74^. Time series data were then filtered into eight narrow frequency bands (with band pass of 2–4, 4–7, 7–10, 10–14, 14–20, 25–45, 50–100 Hz) used in amplitude analysis, and a broadband filtered (1–45 Hz) used in ER analysis and for creating noise covariance matrix (NCM) from broadband filtered resting-state data. NCMs were used to construct MNE inverse operator with regularization constant of 0.05 for filtered single trial MEG time series. The 6800–8000 source dipole time series were collapsed into time series of 402 cortical parcels obtained from Destriux atlas by using fidelity weighted averaging^75^.

### MEG data analysis

In T1 all trials with correct answers were combined. Stimulus period which did not include motor-response-related activity were used for data-analysis. In T2, trials where no responses were given were used to avoid confounding effects of motor-response-related signals. In T1, 134 ± 10 (mean ± SD) trials for amblyopic patients (*N* = 11) and 140 ± 3 for healthy controls (*N* = 11) and in T2, 88 ± 11 trials for amblyopic patients (*N* = 13) and 96 ± 4 for healthy controls (*N* = 11) were analysed. Analysis of ERs and oscillation amplitudes was carried out as in Refs.^36,38,39^ and is described here only briefly. Collapsed parcel wise inverse estimates X_F,P,*r*_(*t*,*f*) of single trials *r*, *r* = 1… *n*_*s*_, were used for cortex wide mapping of *ER*s and induced amplitude (*A*) dynamics. Hilbert transform was used to obtain to amplitude time series from narrow-band filtered data *A*(*t*,*f*), by $A = n_{s}^{ - 1} \sum\nolimits_{{\text{r}}} {\left( {\left| {{\text{x}}_{{{\text{F}},{\text{P}},a}} } \right|} \right)}$. Time series were then collapsed in 100 ms windows with a 50 ms overlap. Broad-band filtered data was used to compute *ER*(*t*,*f*) by averaging real parts of time series so that $ER = n_{s}^{ - 1} \sum\nolimits_{r} {\left[ {{\text{Re}} (x_{{{\text{F}},{\text{P}},a}} )} \right]}$. Oscillation amplitudes and ERs were estimated for each 402 cortical parcels and for oscillation amplitudes also for each narrow-band frequency and averaged separately for each task and condition for each subject.

### Group statistics

Statistical testing of ERs and oscillations amplitudes was carried out after subtracting the mean baseline 0.3–0.05 s before stimulus onset and collapsing data into 202 Destrieux parcels. Group statistics were performed separately for each frequency, time window and cortical parcel. Significant difference of neuronal activity between stimuli and baseline as well as between the two eyes was estimated with paired *t*-test (threshold *p* < 0.05 or *p* < 0.01). False discovery rate (FDR) was reduced by removing as many of the least significant positive and negative tail findings as predicted by the alpha-level of 0.05 or 0.01. The number of predicted findings by alpha-level is Alpha x N cortical parcels for each time and frequency bin. To test for the hemispheric specific effects, parcels were switched between hemispheres when AE was the left eye, but as these data were very similar to original, it is not shown. Welch’s *t*-test assuming dissimilar distributions between test cohorts was used to compare neuronal activity between patient and control groups. Raw *p*-values of the ER and amplitude tests are provided as Supplementary Information.

To estimate whether reduced neuronal activity for AE was correlated with deficits in visual acuity, we first identified maximum values for the difference of oscillation amplitudes (2–10 Hz) and ERs between FE and AE in the time-window of 0.1–0.4 s from stimulus onset in two a priori determined ROIs, V1/V2 and a ROI including LOC and V4–V8. We then estimated the correlation of these values with the visual acuity (LogMAR) values in AE (or non-DE) using the Pearson correlation coefficient. The statistical values were corrected for multiple comparisons with Bonferroni corrections (6 tests).

### Visualization

Averaged ER and oscillation amplitudes were visualized as fraction of parcels having statistically significant positive and/or negative effect out of all parcels (P+/P−). To reveal brain areas accounting for the most prominent effects, these data were visualized on an inflated flattened cortical surface, which enables presentation of all data, and specifically of all visual cortical regions distributed into medial and lateral surfaces. To facilitate interpretation, we marked boundaries of functions visual systems as in Refs.^49–51^ (see Supplementary Materials and Methods).

## Supplementary Information

Supplementary Information 1.

Supplementary Information 2.

## Data Availability

The ethical permission does not allow the public sharing of the data. Data can be accessed upon reasonable request from the authors.
