# Supplementary material for: Reduced evoked activity and cortical oscillations are correlated with anisometric amblyopia and impairment of visual acuity
Source: Sci Rep. 2021 Apr 15;11:8310. doi: 10.1038/s41598-021-87545-9 (PMC8050307; doi:10.1038/s41598-021-87545-9)
Supplement: Supplementary file 1 — Supplementary Information 1. [file 41598_2021_87545_MOESM1_ESM.pdf]

Reduced evoked activity and cortical oscillations are correlated with anisometric amblyopia and impairment of visual acuity

Hanna Julku, Santeri Rouhinen, Henri J. Huttunen, Laura Lindberg, Johanna Liinamaa, Ville Saarela, Elina Karvonen, Sigrid Booms, Jyrki P. Mäkelä, Hannu Uusitalo, Eero Castrén, J. Matias Palva, and Satu Palva

SUPPLEMENTARY MATERIALS AND METHODS

Inclusion and exclusion criteria of amblyopic patients

In the trial, inclusion criteria were as follows: age 18–60 years, male or female, diagnosis of moderate to severe (0.3–0.6 logMAR difference to >0.6 logMAR difference) amblyopia due to myopic or hyperopic anisometropia or congenital esotropia. Amblyopia was considered to be of anisometropic if both refractive error was at least 1 D of anisometropia (spherical equivalent) and no strabismus had been diagnosed in childhood. In contrast, we excluded subjects with another forms of strabismus (than congenital esotropia) as the primary reason for amblyopia, history of any amblyopia therapy in the previous 2 years before the screening visit, any eye surgery less than 6 months before the screening visit, observed off-fixation in ophthalmological examination (extra-foveal/eccentric fixation) or other ophthalmological pathologies. Therefore, only amblyopic subjects with anisometropia or congenital strabismus (without binocular vision) were included due to a potential risk of intractable diplopia in individuals with acquired strabismus and amblyopia treated in adulthood [1]. All demographic data, medical history, physiological and ophthalmologic data were obtained as part of the trial.

Clinical Eye examination

Crowded near visual acuity was assessed using crowded Landolt C ring chart booklet at a distance of 40 cm and defined by the smallest line in which the subject correctly identified at least 8 of 12 letters (≥66.7%). Contrast sensitivity was determined using a Pelli-Robson chart at a distance of 1 m (charts A and B). The contralateral eye was occluded during testing and charts were switched between eyes. A slit lamp examination was performed at the initial visit to assess the structure of the eyes.

Binocularity was examined using the Bagolini striated glass test before monocular testing [2]. Lens striations were placed at 135° before the right eye and 45° before the left eye using lorgnette frames. The Bagolini striated glass test setup allows each eye to receive the same fusible image with each fixation streak oriented perpendicular to the striations and 90° away from the other eye. The test enables the evaluation of simultaneously perceived images with a minimal dissociative effect and it was performed at near (33 cm) and distance (4 m) under normal lighting conditions. Binocularity was categorized as suppression (1 light and only 1 line were seen), normal fusion (binocular single vision, BSV; 2 lines were seen as X and 1 light at the centre), anomalous retinal correspondence (ARC; harmonious if 1 light and 2 lines were seen, but one of the lines was broken due to foveal suppression, or inharmonious if 1 light and 2 lines were seen, but the lines did not cross at the centre where the light was located) or diplopia (2 lights and 2 lines were seen). None of the subjects had diplopia in the current study.

Task 2 Change detection task

Complex visual objects were designed manually prior to the experiment to be generated at run-time so that the contours defining the layers of the objects were defined by a bank of additive and multiplicative simple algebraic functions. The objects were randomly picked from a pool of 15 distinct object shapes, realized with randomized hues and variable sizes, and presented in random locations of a 2 x 2 grid around a central fixation point. The objects' centre was locked to the centre of the grid cell and during the presentation time, the curvature of the objects changed along their longitudinal axis and they rotated around their centre. These motions were generated by moving these objects along curvilinear paths as described by [3] but with the translational movement eliminated by the locking of the object's centre to the grid cell. The object hues and colour maps for the Perlin-noise background images of task blocks were automatically generated from the pre-made palettes and the differences in contrast values were minimal between the trials.

Definition of functional visual systems

V1–V2 (calcarine sulcus, cuneus, lingual gyrus, occipital pole), LOC (superior occipital gyrus /sulcus), V4–V8 (posterior collateral sulcus, medial occipito temporal sulcus, middle occipital gyrus /sulcus), ventral stream

(anterior collateral sulcus, inferior temporal gyrus / sulcus, occipito temporal gyrus /sulcus, superior temporal sulcus / gyrus); dorsal stream (intraparietal sulcus, superior parietal gyrus, parieto occipital sulcus).

References

[1] Newsham D, O'Connor AR. Assessment of the Density of Suppression to Identify Risk of Intractable Diplopia in the United Kingdom. Strabismus 2016 Jun;24(2):45-50.

[2] Bagolini B. Sensorial anomalies in strabismus. (suppression, anomalous correspondence, amblyopia). Doc.Ophthalmol. 1976 Apr 28;41(1):1-22.

[3] Huttunen HJ, Palva JM, Lindberg L, Palva S, Saarela V, Karvonen E, et al. Fluoxetine does not enhance the effect of perceptual learning on visual function in adults with amblyopia. Sci.Rep. 2018 Aug 27;8(1):12830,018-31169-z.

Comparison	T1	T2
Performance, AE vs. FE	No difference	Worse for AE
ER, AE vs. FE	No difference	Smaller for AE
Amplitude, AE vs. FE	3–14 Hz stronger for AE<br>15–30 Hz stronger for FE	3–10 Hz stronger for AE<br>10–30 Hz stronger for FE
ER, FE vs. DE	Smaller for FE	No difference
Acuity with ER correlation, AE	Not correlated	Not correlated
Acuity with amplitude correlation, AE	Correlated in low f condition<br>Not correlated in high f condition	Correlated

Supplementary Table 1. An overview of the findings. AE: amblyopic eye, FE: fellow eye, ER: evoked response, DE: dominant eye.

ID	Age	Sex	Contrast sensitivity	LogMAR VA AE/Non-DE	LogMAR VA FE / DE	Anisome-tropia (D)	Diagnosis	AE/ Non-DE	Included in T1	In T2
**P01**	42	F	1.95	0.36	-0.08	1.75	mh, ss	left	+	+
**P02**	35	F	1.95	0.76	-0.12	2.75	mh, ss	right	+	+
**P03**	46	F	1.95	0.76	-0.12	1.25	mh, ss	right	+	+
**P04**	37	F	1.95	0.3	-0.08	4.25	mh	right	-	+
**P05**	32	M	1.95	0.54	-0.1	2.63	mh	left	-	+
**P06**	49	F	N/A	0.3	-0.2	2.5	mh	right	+	+
**P07**	47	F	0	1.08	-0.04	3.5	mh, ce, ss	right	+	+
**P08**	46	M	1.95	0.56	-0.2	2.5	mh	left	+	-
**P09**	37	M	1.95	0.34	0	1.75	mh	left	+	+
**P10**	37	M	1.95	0.44	0	0.25	mh, as	left	+	-
**P11**	33	M	1.95	0.46	-0.18	2.5	mh, ms	right	+	+
**P12**	45	M	1.95	0.68	-0.12	1.25	ce	right	+	+
**P13**	53	M	1.95	0.6	-0.14	3.75	mh	left	+	+
**P14**	57	M	1.65	1.04	0.04	0.75	ce, mh	left	-	+
**P15**	57	M	1.95	0.36	-0.18	3.75	mh	left	-	+
**C01**	40	M	N/A	-0.01	0	N/A	N/A	left	+	+
**C02**	31	M	N/A	-0.12	-0.22	N/A	N/A	left	+	+
**C03**	30	M	N/A	-0.16	-0.2	N/A	N/A	left	+	+
**C04**	25	M	N/A	-0.12	-0.22	N/A	N/A	left	+	+
**C05**	30	M	N/A	-0.22	-0.22	N/A	N/A	left	+	+
**C06**	25	M	N/A	-0.2	-0.2	N/A	N/A	left	+	+
**C07**	49	F	N/A	-0.1	-0.1	N/A	N/A	left	+	+
**C08**	27	F	N/A	-0.12	-0.2	N/A	N/A	right	+	+
**C09**	26	M	N/A	-0.06	-0.12	N/A	N/A	right	+	+
**C10**	41	M	N/A	-0.02	-0.1	N/A	N/A	left	+	+
**C11**	39	F	N/A	-0.14	-0.18	N/A	N/A	right	+	+
**P ave.**	43.5		1.8	0.572	-0.10	2.34				
**P SD**	8.3		0.5	0.251	0.08	1.13				
**C ave.**	33.0		N/A	-0.115	-0.16	N/A				
**C SD**	8.0		N/A	0.067	0.07	N/A				

Supplementary table 2. Demographic and ophthalmologic information of the patient and control cohorts. P in ID refers to patients, C to controls. Average and standard deviation values for patients and controls are presented on the four last rows. Contrast sensitivity is reported for the patients' amblyopic eye (AE) as the contrast sensitivity of the fellow eye (FE) was normal in all patients (cut-off level of 1.95 for a monocular test at 1 m distance). Visual acuities (VA) are presented for the AE and FE of the patients and for the non-dominant eye (non-DE) and the dominant eye (DE) of the controls. On a logMAR scale, smaller VA values indicate better vision. Therefore, 0 refers to normal vision (Snellen VA of 1.0) and the cut-off level 1.3 for blindness (Snellen VA of 0.05). Negative logMAR values indicates VA is better than normal. All included patients had moderate to severe amblyopia with logMAR VA of 0.3–0.6 or >0.6 in the AE (corresponding Snellen VA of 0.25-0.5 or <0.25). Key words: VA= visual acuity; D=diopter; ce=congenital esotropia; mh=myopic/hyperopic anisometropia; ss=secondary strabismus; as=astigmatic anisometropia; ms=microstrabismus; N/A denotes that the measures were not obtained.

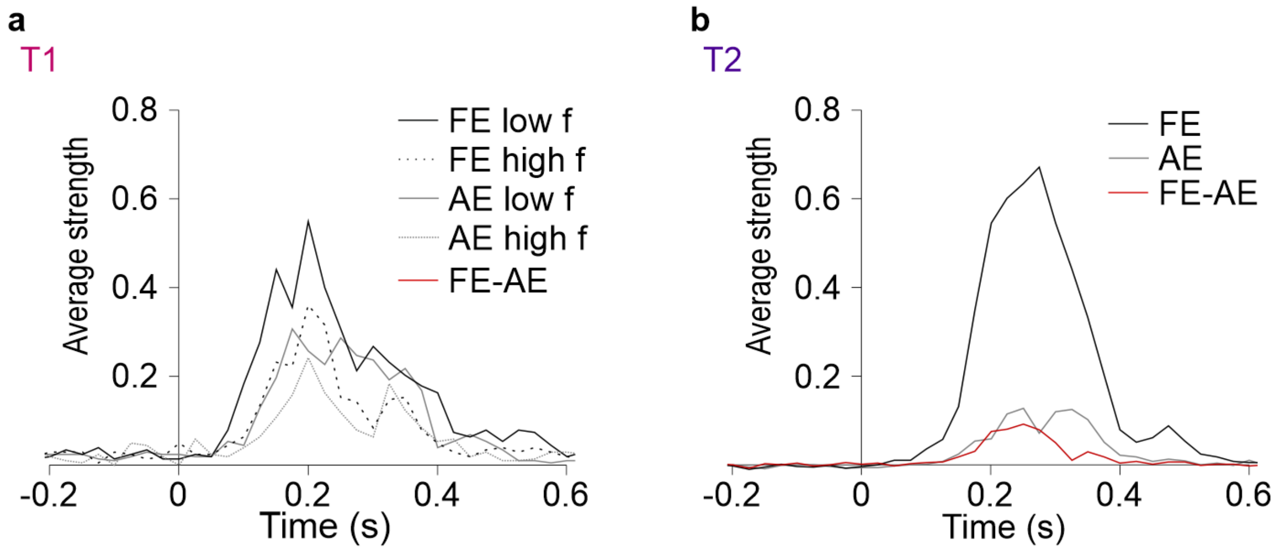

Supplementary Figure 1. ERs are reduced for AE in T2. a) Average strength of parcels showing significant ER compared to the baseline. Black line denotes fraction of parcels for FE and grey line that for AE (*t*-test, threshold $p < 0.05$, FDR corrected). **b**) Same as a but for T2. Red line denotes the difference of average strength of parcels where FE and AE ER was significantly different (*t*-test, threshold $p < 0.05$, FRD corrected).

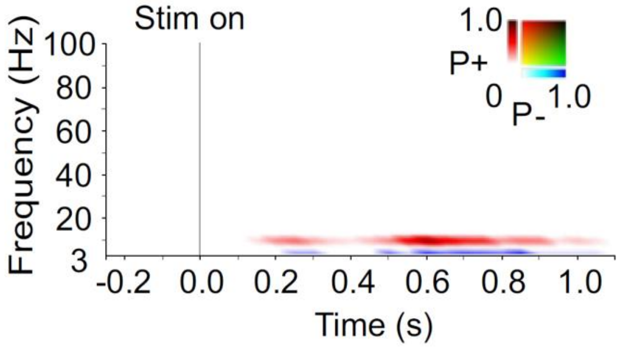

Supplementary Figure 2. Task dependent modulations of oscillation amplitudes in T1. The difference of oscillation amplitudes between stimuli presented to the FE and AE in the T1 for the difference in between low and high spatial frequency stimuli (*t*-test, $p < 0.01$). Red indicates the FE-AE difference was larger for the low-spatial frequency stimuli, and blue colours for the high-spatial frequency stimuli.

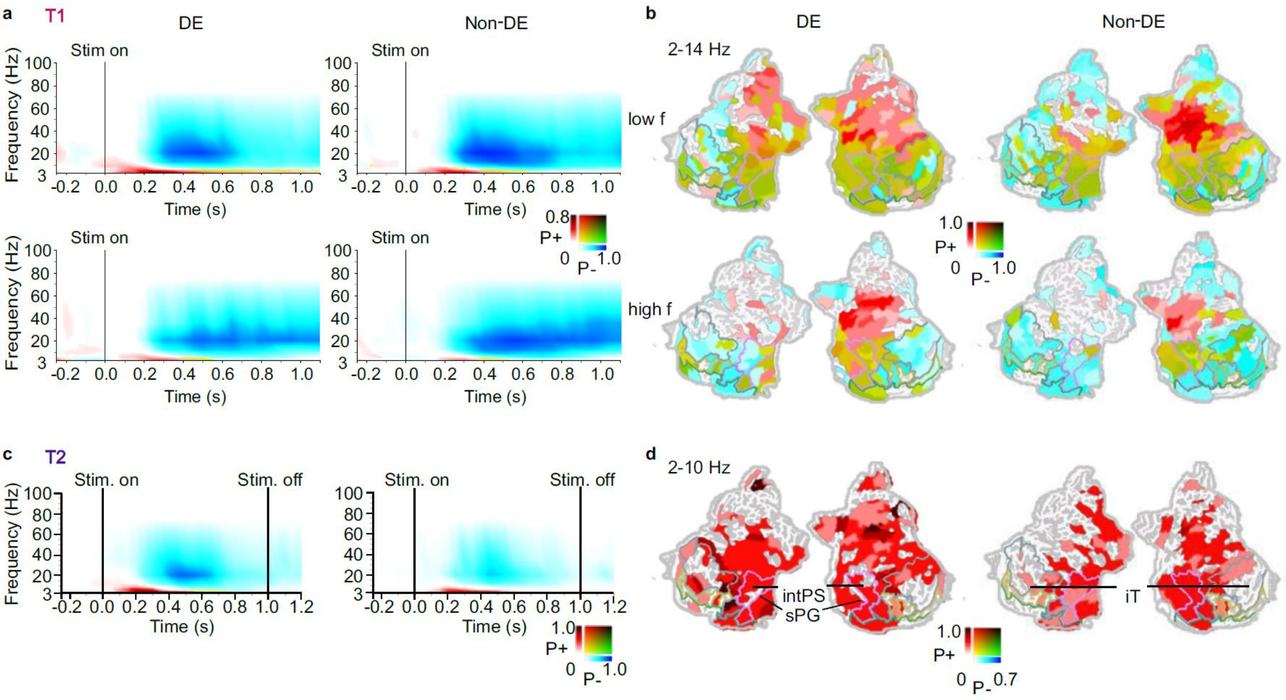

Supplementary Figure 3. Task dependent modulations of oscillation amplitudes in control subjects. TFRs of oscillation amplitudes separately for the dominant eye (DE) and the non-DE (*t*-test, $p < 0.05$, corrected) in **a**) discrimination task, T1 and **c**) change detection task, T2. **b**) Cortical localization of amplitude modulations over 2–14 Hz and 0.25–0.3 s (*t*-test, $p < 0.05$, corrected) of T1. **d**) Cortical localization of amplitude modulations over 2–10 Hz and 0.25–0.3 s (*t*-test, $p < 0.01$, corrected) for T2. Abbreviations: intPS intraparietal sulcus; sPG superior parietal gyrus; iT inferior temporal cortex.
